# Evaluation of selected South African ethnomedicinal plants as mosquito repellents against the *Anopheles arabiensis *mosquito in a rodent model

**DOI:** 10.1186/1475-2875-9-301

**Published:** 2010-10-28

**Authors:** Rajendra Maharaj, Vinesh Maharaj, Marion Newmarch, Neil R Crouch, Niresh Bhagwandin, Peter I Folb, Pamisha Pillay, Reshma Gayaram

**Affiliations:** 1 South African Medical Research Council, 491 Ridge Road, Overport, Durban 4001, South Africa; 2 Biosciences, CSIR, P.O. Box 395, Pretoria 0002, South Africa; 3 Ethnobotany Unit, South African National Biodiversity Institute, PO Box 52099, Berea Road 4007/School of Chemistry, University of KwaZulu-Natal, Howard College Campus, Durban 4041, South Africa; 4 South African Medical Research Council, P.O. Box 19070, Tygerberg 7505, South Africa

## Abstract

**Background:**

This study was initiated to establish whether any South African ethnomedicinal plants (indigenous or exotic), that have been reported to be used traditionally to repel or kill mosquitoes, exhibit effective mosquito repellent properties.

**Methods:**

Extracts of a selection of South African taxa were tested for repellency properties in an applicable mosquito feeding-probing assay using unfed female *Anopheles arabiensis*.

**Results:**

Although a water extract of the roots of *Chenopodium opulifolium *was found to be 97% as effective as DEET after 2 mins, time lag studies revealed a substantial reduction in efficacy (to 30%) within two hours.

**Conclusions:**

None of the plant extracts investigated exhibited residual repellencies >60% after three hours.

## Background

Despite ongoing efforts to control the disease, malaria still remains a serious public health problem in about 90 countries worldwide. On a global scale, malaria causes 300-500 million cases and results in 1.5-3 million deaths annually. Of these, approximately 80% of cases occur on the African continent. In the Southern Africa Development Community (SADC) mortality ranges from 0 to 128 per 100,000 population [1].

This large-scale threat to human health is transmitted by anopheline mosquitoes. These vectors feed between dusk and dawn, mostly inside houses whilst residents are asleep. However, due to behaviour modification by both vector and host, mosquitoes increasingly feed outdoors, so avoiding the insecticides sprayed on inner walls of houses in the course of vector control programmes [2].

In order to provide protection to target individuals who are outdoors during the period of active feeding, repellents are used. Repellents are applied to exposed skin, especially the arms and legs, to protect individuals against mosquitoes seeking a blood meal. The most successful active ingredient of several skin-applied mosquito repellent products is N,N-diethyl-meta-toluamide (DEET) [3]. Although presently highly effective, there have been some concerns of DEET's adverse side effects when used in high concentrations [4]. In southern Africa, limited work has been conducted on the search for natural repellent products. Although the value of numerous synthetic compounds have been investigated for repellency against *Anopheles gambiae *[5], there have been very few studies investigating the use of plant-derived chemicals as repellents of vectors of malaria.

Apart from employing antiplasmodials [6] and insecticidal interventions, communities in Africa have historically employed traditional methods to ward off bothersome mosquitoes, even if the association between malaria and this vector has not been made. Different methods include burning of cow dung or certain plants, or the placement of specific plant parts in and around the sleeping area. Plants have long been recognized as having insect repellent properties. In endemic malaria areas, plants have been crushed or used whole to provide protection against mosquitoes [7], or burned to this effect [8].

As part of a national programme to identify new products from medicinal plants to combat malaria, this study was initiated to establish whether any indigenous or naturalized South African ethnomedicinal plants that have been reportedly used traditionally to repel or kill mosquitoes exhibit effective mosquito repellent properties. Accordingly, extracts of a selection of a number of such taxa were tested for their mosquito repellency properties in a suitable bioassay, which assesses the potency of the test substance when applied topically to the skin.

## Methods

### Selection and collection of plant material

A survey of relevant literature on ethnomedicinal plants used in East and Southern Africa revealed that a number of taxa have been reported to be used as mosquito repellents, or to repel or kill other invertebrates. However, given the limited quality of documented data available, it was decided to not make a distinction between insect repellents and insecticides (both larva- and adulticides), but to rather consider a pool of plants with anti-insecticidal activity. This decision was in part based on earlier observations [9] that some plants possessing repellent properties are also insecticidal. The converse could be true. In order to select the most relevant taxa, all were ranked following the application of weighted criteria, principally ethnobotanical and chemotaxonomic (including such elements as popularity in ethnomedicinal trade, reports on insecticidal and/or mosquitocidal application, reports on insect and/or mosquito repellent application, and the known presence and diversity of repellent/insecticidal constituents in the family to which it belongs). Higher weighting was provided to plants indigenous to the *Flora of southern Africa *region. A similar semi-quantitative selection method has previously been applied to identify and rank anti-plasmodial plant candidates from South Africa [6].

From the ranked list selected plants were collected throughout South Africa. Different plant parts, namely, leaves, root, stem, fruit, flowers, seeds, twigs and bark, and combinations of the above were sourced to generate extracts. In some instances, extracts were made of the whole plant. The plant organ(s) selected for extract preparation was based largely on availability at the time of collection.

The identity of plant material was determined at the National Herbarium of South Africa (PRE) where voucher specimens have been lodged.

### Extract preparation

Plant samples were separated into different components and dried in an oven at 30-60°C. The drying time and temperature varied depending on the nature of the plant part. Dried plant material was ground to a coarse powder using a hammer mill and stored at ambient temperature prior to extraction. For each extraction procedure, 100-500 g of powdered plant material was sequentially extracted, with cold dichloromethane (DCM), DCM/methanol (MeOH) (1:1), MeOH and purified water. Organic extracts were concentrated by rotary vacuum evaporation below 45°C and then further dried *in vacuo *at ambient temperature for 24 h. The aqueous extracts were concentrated by freeze-drying. All dried extracts were stored at -20°C.

### Animal preparation

Repellent activity was assessed by topical application of the test substance to the ventral surface of test rodents, and subsequent exposure of the treated area to unfed female mosquitoes. The number of bites relative to the untreated negative control was recorded, and the repellency percentage determined. The standard WHO guidelines were adapted for use in this trial [10].

The rodent, *Mastomys coucha *was used for the screening of the extracts. Ethical approval for the use of live animals in this study was obtained from the Ethics Committee of the South African Medical Research Council. Animals were put into groups of four, and each plant extract was tested on two animals, whilst the remaining two animals were used as negative and positive controls respectively. Carriers for the extracts, either acetone or distilled water, were used as the untreated negative control whereas concentrated, laboratory grade DEET was used as the positive control. Crude plant samples were dissolved in either acetone or distilled water depending on their initial extraction procedure thus forming a 10 mg/ml solution. DCM and DCM/MeOH extracts were reconstituted in acetone whereas aqueous extracts were made up in distilled water.

Adult rodents were weighed individually, and injected intraperitoneally with a 1 ml solution of sodium pentobarbital (60 mg/l) per 0.225 kg of body weight. Once anaesthetized, rodents were shaved on the ventral surface and 1 ml of plant extract solution applied to the abdomens of each of two rodents. The percentage repellency was taken as the mean of the number of bites relative to the untreated negative control.

### Probing activity assay

Paper cups (500 ml) were modified by replacing the base of the cup with mosquito netting held in place with a rubber band and covering the mouth of the cup with transparent plastic film. Thirty unfed 4-day old *Anopheles arabiensis *females were introduced into the cup and held in contact with the treated ventral surface of each anaesthetized rodent. Mosquito activity was observed through the transparent plastic film. At the end of a two-minute exposure period the number of mosquitoes probing (attempting to feed on the anaesthetized mouse, through the netting) was recorded. For those extracts with protection >80% after 2 mins, the repellency effect was determined hourly for up to three hours post application. The rodent was then returned to the animal facility and allowed to recover from the anaesthetic.

### Results and discussion

A total of 115 plant extracts, derived from 24 taxa representing 14 plant families, were evaluated for their repellency properties, (Table 1). Results have been presented alphabetically by family, genus and species, and thereafter in descending order of repellency effect after the two-minute exposure period.

**Table 1:** Two minute mosquito repellency screening results for extracts of South African ethnomedicinal plants.

Family	Plant species	Voucher number	Plant Part	Extraction	Repellency (%)
**Apiaceae**	**Apiaceae**	EN00994	Whole plant	DCM/MeOH (1:1)	55
	*Alepidea amatymbica *Eckl. & Zeyh.				
			Whole plant	Water	23
**Asphodelaceae**	*Aloe greatheadii *Schönland var. *davyana *(Schönland) Glen & D.S. Hardy	EN00021	Leaves	DCM	55
		EN00021	Leaves	DCM/MeOH (1:1)	55
		EN00021	Stem	DCM	53
		EN00021	Leaves	Water	43
		EN00021	Leaves	DCM	36
		EN00021	Stems	DCM/MeOH (1:1)	33
		EN00021	Stems	MeOH	31
		EN00021	Stems	Water	27
	*Aloe ferox *Mill.	EN00538	Fruit	DCM	68
		BP00469	Whole plant	DCM/MeOH (1:1)	58
		EN00538	Roots	DCM	56
		EN00538	Stem	DCM/MeOH (1:1)	52
		EN00538	Roots	DCM/MeOH (1:1)	50
		EN00538	Leaves	DCM/MeOH (1:1)	40
		EN00538	Leaves	DCM	33
		EN00538	Stems	DCM	33
		EN00538	Roots	Water	31
		EN00538	Stems	Water	31
		BP00469	Whole plant	Water	30
		EN00538	Fruit	Water	26
		EN00538	Leaves	Water	26
		EN00538	Fruit	DCM/MeOH (1:1)	21
**Asteraceae**	*Bidens pilosa L.	EN00001	Leaves	DCM/MeOH (1:1)	43
		EN00001	Leaves	DCM	40
		EN00001	Leaves	Water	31
		EN00001	Leaves	MeOH	30
	*Chromolaena odorata (L.) R.M.King & H.Rob.	EN00052	Leaves	MeOH	80
		EN00052	Leaves	DCM	76
		EN00052	Whole plant	MeOH	68
		EN00052	Leaves	DCM/MeOH (1:1)	67
		EN00052	Whole plant	DCM	61
		EN00052	Whole plant	DCM/MeOH (1:1)	57
		EN00052	Flowers	MeOH	50
		EN00052	Leaves	Water	46
		EN00052	Whole plant	Water	31
		EN00052	Flowers	Water	25
		EN00052	Flowers	DCM	18
		EN00052	Flowers	DCM/MeOH (1:1)	15
	*Eclipta prostrata (L.) L.	DS00616	Whole plant	Water	67
		DS00616	Whole plant	DCM/MeOH (1:1)	20
	*Litogyne gariepina *(DC.) Anderb.	EN00213	Roots	DCM	83
		EN00213	Roots	Water	30
		EN00213	Roots	DCM/MeOH (1:1)	28
**Buddlejaceae**	*Nuxia floribunda *Benth.	BP00669	Leaves	DCM/MeOH (1:1)	85
			Leaves	Water	28
**Chenopodiacae**	*Chenopodium ambrosioides L.	BP00545	Leaves	DCM/MeOH (1:1)	87
		BP00545	Twigs	DCM/MeOH (1:1)	31
		BP00545	Leaves	DCM/MeOH (1:1)	25
	*Chenopodium opulifolium Schrad. ex W.D.J.Koch & Ziz	DS00390	Roots	Water	97
		DS00390	Stems	Water	67
		DS00390	Roots	DCM/MeOH (1:1)	33
		DS00390	Leaves	DCM/MeOH (1:1)	33
		DS00390	Stems	DCM/MeOH (1:1)	30
		DS00390	Leaves	Water	20
**Euphorbiaceae**	*Croton pseudopulchellus *Pax	HV00052	Leaves	DCM/MeOH (1:1)	33
		HV00052	Leaves	Water	25
	*Spirostachys africana *Sond.	EN00346	Stems	DCM/MeOH (1:1)	48
		EN00346	Stems	Water	33
		EN00346	Stems	DCM	28
		BP00230	Leaves and twigs	DCM/MeOH (1:1)	18
**Fabaceae**	*Dichrostachys cinerea *(L.) Wright & Arn. subsp. *africana *Brenan & Brummitt	EN00101	Leaves	MeOH	67
		EN00101	Stems	DCM/MeOH (1:1)	65
		EN00101	Stems	DCM	50
		EN00101	Leaves	Water	46
		EN00101	Stems	Water	33
		EN00101	Leaves	DCM/MeOH (1:1)	33
		EN00101	Leaves	DCM	28
	*Mundulea sericea *(Willd.) A.Chev.	BP00043	Leaves	DCM/MeOH (1:1)	76
		BP00043	Leaves	DCM	72
		BP00043	Leaves	MeOH	48
		BP00043	Leaves	Water	26
	*Philenoptera violacea *(Klotzsch) Schrire (syn.	MM00019	Stems	DCM	55
	*Lonchocarpus capassa *Rolfe)				
		MM00019	Leaves	DCM/MeOH (1:1)	31
		MM00019	Stems	Water	30
		MM00019	Leaves	DCM/MeOH (1:1)	30
		MM00019	Seeds	Water	28
		MM00019	Roots	DCM/MeOH (1:1)	28
		MM00019	Leaves	Water	27
		MM00019	Bark	DCM/MeOH	26
		MM00019	Roots	Water	21
		MM00019	Bark	Water	20
**Lamiaceae**	*Leucas martinicensis *(Jacq.) R.Br.	EN00183	Leaves	DCM	86
		EN00183	Leaves	DCM/MeOH (1:1)	52
		EN00183	Leaves	Water	26
	*Mentha longifolia *(L.) Huds.	EN00296	Whole plant	DCM/MeOH (1:1)	56
		EN00296	Whole plant	DCM	55
		EN00296	Whole plant	Water	18
	*Plectranthus laxiflorus *Benth.	EN00195	Leaves	Water	55
		EN00195	Leaves	DCM	53
		EN00195	Leaves	DCM/MeOH (1:1)	38
**Malvaceae**	*Sida cordifolia *L.	FP00185	Whole plant	DCM/MeOH (1:1)	87
		FP00185	Whole plant	Water	40
**Meliaceae**	*Melia azedarach L.	DS00379	Leaves	DCM/MeOH (1:1)	60
		DS00379	Leaves	Water	33
**Pedaliaceae**	*Ceratotheca triloba *(Bernh.) Hook.f.	BP00646	Leaves	DCM/MeOH (1:1)	50
		BP00646	Twigs	DCM/MeOH (1:1)	40
		BP00646	Twigs	Water	31
		BP00646	Leaves	Water	30
		BP00646	Fruit	Water	26
**Rutaceae**	*Clausena anisata *(Willd.) Hook.f. ex Benth.	DS00090	Leaves	DCM/MeOH (1:1)	56
		DS00090	Stems	DCM/MeOH (1:1)	31
		DS00090	Stems	Water	31
		DS00090	Leaves	Water	16
**Solanaceae**	*Datura stramonium L.	EN00769	Leaves	DCM/MeOH (1:1)	81
		EN00769	Fruit	DCM/MeOH (1:1)	40
		EN00769	Leaves	Water	30
		EN00769	Fruit	Water	23
**Vitaceae**	*Cissus cornifolia *(Baker) Planch.	MM00008	Stems	DCM/MeOH (1:1)	45
		MM00008	Roots	DCM/MeOH (1:1)	34
		MM00008	Leaves and flowers	DCM/MeOH (1:1)	31
		MM00008	Roots	Water	30
		MM00008	Stems	Water	25
		MM00008	Leaves and flowers	Water	25

* exotic to South Africa

Stringent criteria were used to assess the biological activity, in order to determine their potential as plant-derived repellents. Since these were crude plant extracts, a mosquito repellency effect of 80% was considered significant for further investigation. Seven samples from seven taxa showed ≥80% repellency, whereas the remaining 109 samples gave between 15 and 76% efficacy relative to the negative control. However, a repellent is only effective if it has a long-lasting effect. Since DEET is known to have an eight hour effect, for the purposes of these investigations, substantial repellency (>80%) after three hours was taken to be an indication of high activity worth of further investigation. To ensure that the highly active extracts maintained a constant repellent effect over a period of three hours, five of the seven samples indicating repellency ranging from 83 to 97% were subjected to a time lag trial (Table 2). This further evaluation was deemed an important requirement in considering their further industrial development, as market competitors of DEET should necessarily exhibit repellency over an extended period.

**Table 2:** Time lag repellency tests using a selection of active extracts from initial two minute trials

Family	Plant species	Plant part	Extraction	Repellency%		
				(minutes post application)		
				60	120	180
Asteraceae	*Litogyne gariepina*	Roots	DCM	75	52	57
Buddlejaceae	*Nuxia floribunda*	Leaves	DCM/MeOH(1:1)	43	42	35
Chenopodiaceae	*Chenopodium opulifolium*	Roots	Water	97	17	30
Lamiaceae	*Leucas martinicensis*	Leaves	DCM	60	57	49
Malvaceae	*Sida cordifolia*	Whole Plant	DCM/MeOH(1:1)	40	23	10
Negative control			Distilled water	10	20	20
Negative control			Acetone	17	20	33
Positive control			DEET	100	100	100

From the time lag studies, it was found that post-application, the most active extracts rapidly lost activity. Compared to the positive control, DEET, these extracts were not long-lasting.

None of the extracts satisfied the 80% repellent activity cut off point after three hours. The most active species, *Chenopodium opulifolium *(Chenopodiaceae) was found only 30% effective relative to the negative control after three hours post application (Table 2). However, as only crude extracts were assayed in all repellency trials it is possible that active constituents isolated from roots of *C. opulifolium *could show prolonged repellency.

In considering the plant families used in this study, two of the seven most active extracts belong to the Asteraceae family. *Artemisia absinthium *has been shown to possess tick repellent properties [9]; the authors found that this plant synthesized volatiles that exhibited insecticidal properties. Previous studies have shown that plants belonging to the Fabaceae [11,12] are noted for their larvicidal activity. Park *et al *[13] found that monoterpenes from the Lamiaceae could be used to repel mosquitoes of the genus *Culex*, Similarly, octacosane derived from *Moschosma polystachyum *(Lamiaceae) was effective in repelling *Culex *[14].

Globally, an enormous amount of work has been completed in trying to develop mosquitocidal compounds from plants. The use of plants as larvicides or adulticides is not a novel concept. A number of the insecticides currently used for malaria control are products of plants [15]. Limited research has been completed on the search for natural repellent products *Apium graveolens *(Apiaceae) is capable of repelling *Aedes*, *Anopheles *and *Mansonia *species [16], whilst the essential oils of ginger, *Zingiber officinale *(Zingiberaceae) and rosemary, *Rosmarinus officinalis *(Lamiaceae) were found to be repellent towards *Anopheles stephensi, Aedes aegypti *and *Culex quinquefasciatus *[17]. However, whilst in these reports the percentage of mosquitoes repelled were lower than observed in most plants reported in the present study, the longevity of effect was greater. Nevertheless, these studies used vectors that are not found in southern Africa.

A study conducted in western Kenya [8] has shown limited efficacy of traditionally used mosquito repellents; plants were either burnt or used whole, resulting in a maximum repellency effect of 52% against *An. gambiae*.

Numerous studies have been completed where chemicals have been tested for repellency effect against *An. gambiae *[18,19]. There is limited information available on the use of plant-derived chemicals as repellents of mosquitoes, especially *An. gambiae.*

Although all extracts demonstrated some bioactivity (15-97%), none of the extracts tested displayed repellency comparable to DEET after a prolonged period. Even though cognisance was taken of the fact that these were crude plant extracts none of them exceeded the minimum acceptable repellency level (80%) during time lag studies. Organic extracts of the plants in general showed more potent repellency relative to their corresponding aqueous extract. This indicates that the lower molecular weight compounds may be contributing to the repellency properties rather than the macromolecules generally found in aqueous based extracts. Exceptions were the three species *Eclipta prostrata *(Asteraceae), *Chenopodium opulifolium *(Chenopodiaceae) and *Plectranthus laxiflorus *(Lamiaceae), where water extracts were found more potent than their corresponding organic extracts. A further noticeable trend was that the extracts of leaves showed better repellency than extracts of other plant organs from the same species, indicating that volatile components such as essential oils could play a more important role, when formulated appropriately, as plant-sourced mosquito repellents. Notable exceptions to this trend were *Litogyne gariepina *and *Chenopodium opulifolium*, the root extracts of which showed >80% initial repellency.

A limitation of the current study is that volatile compounds were not specifically targeted for mosquito repellency investigation. In this regard, other studies have shown that ethyl acetate extracts of selected plants significantly reduce probing activity of *Aedes aegypti *[20].

## Conclusions

None of the plants investigated in the current study proved to be a potential candidate for the development of commercial repellents from crude extracts due to their rapid loss of efficacy.

## Competing interests

The authors declare that they have no competing interests.

## Authors' contributions

RM was involved in the design of the study and supervising the repellent tests. RG carried out the experiments and was involved in the interpretation of the results. NRC and MN rationally selected suitable plant candidates for investigation. VM and PP were responsible for the extract preparation. NB and PF had coordinated and provided scientific inputs into the entire study. All authors read and approved the final manuscript.
